# Hepatic metastatic paraganglioma 12 years after retroperitoneal paraganglioma resection: a case report

**DOI:** 10.1186/s12876-019-1061-6

**Published:** 2019-08-08

**Authors:** Zhao-Ru Dong, Yan-Ni Xia, Rui-Yi Zhao, Rui Wu, Kai-Xuan Liu, Kai Shi, Lun-Jie Yan, Cheng-Yu Yao, Yu-Chuan Yan, Tao Li

**Affiliations:** 1 Department of general surgery, Qilu Hospital, Shandong University, Jinan, 250012 People’s Republic of China; 2 Department of Operating Room, Qilu Hospital, Shandong University, Jinan, 250012 China; 3 Department of Clinical Laboratory, Shandong First Medical University, Jinan, 250012 China

**Keywords:** Paraganglioma, Metastasis, Liver, Anemia, Transarterial chemoembolization

## Abstract

**Background:**

Paragangliomas, also known as chemodectomas, are rare tumors arise from chemoreceptor tissue, and most commonly locate at the bifurcation of the common carotid, the jugular foramen, aortic arch, and retroperitoneum. Paragangliomas generally are considered to be benign tumors, and rarely produce local or distant metastases. Metastasis to liver is extremely rare.

**Case presentation:**

We report the case of a 39-year-old woman, who had undergone resection of a retroperitoneal paraganglioma at her local hospital for 12 years. She was referred to our hospital for further evaluation of a hepatic mass, which was misdignosed as hepatocellular carcinoma (HCC) and was treated by transarterial chemoembolization (TACE) in the local hospital 6 years ago. At admission, CT scan revealed a huge hypervascular mass with many feeding arteries, almost the same size as 5 years ago. Ultrasound-guided biopsy of the liver tumor was performed and immunohistochemical examination confirmed the diagnosis of hepatic metastatic paraganglioma. Though liver metastasis failed to achieve complete response or partial response to TACE treatment, it remained stable without progression during the 7-year follow-up.

**Conclusion:**

Paragangliomas are slow growing tumors and metastasis may develop decades after resection of the primary lesion. Long-term follow-up is necessary, and curative or palliative treatment should be considered to control symptoms, improve life quality, reduce complications and prolong survival.

**Electronic supplementary material:**

The online version of this article (10.1186/s12876-019-1061-6) contains supplementary material, which is available to authorized users.

## Backgrounds

Paragangliomas are rare lesions derived from the highly vascularized diffuse neuroendocrine system, with an incidence of 1:30000 [1, 2]. Advances in molecular understanding have led to the recognition that about 30–40% of paragangliomas occur in the setting of germline and somatic mutations [3, 4]. They arise from chemoreceptor tissue, and most commonly locate at the bifurcation of the common carotid, the jugular foramen, aortic arch, and retroperitoneum [5, 6]. Paragangliomas can be functional, but are usually non- functional and present as painless masses.

Because of difficulties in diagnosing malignancy, a considerable proportion of paragangliomas with metastatic potential is regarded as benign at initial presentation, but is identified as malignant during follow-up. It is generally recognised that intra-abdominal paragangliomas have a higher rate of metastases, and the most common distant metastases sites involve the lymph nodes, lungs and bones [7, 8]. Metastasis to liver is extremely rare; therefore treatment and prognosis of hepatic metastatic paragangliomas have never been clearly reported. Here we report the first case of hepatic metastatic paraganglioma that was treated by transarterial chemoembolization (TACE).

## Case presentation

**Fig. 1:**
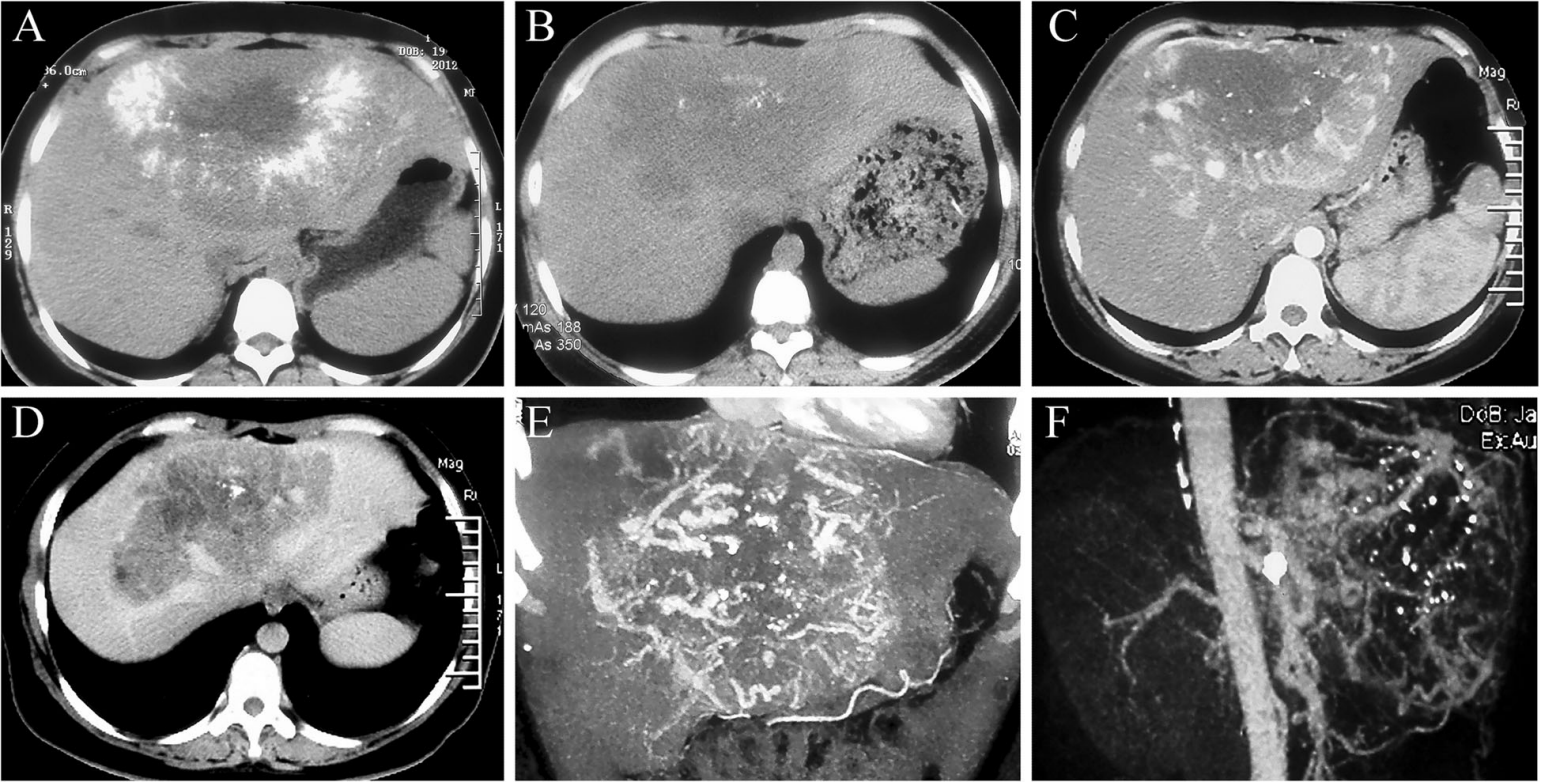
**a**: post-TACE CT scan revealed densely deposited lipiodol inside the tumor; **b**: CT scan revealed a huge solid hypodense mass in the liver; **c**: The lesion was hypodense during the portal phase; **d**: The lesion showed a strong heterogeneous enhancement in the arterial phase; **e**, **f**: Angiography demonstrated a round hypervascular mass with many feeding arteries

A 39-year-old woman was referred to our hospital for further evaluation of a hepatic mass, which was discovered incidentally 6 years ago in the local hospital during physical examination for anemia and fatigue. At that time, the tumor was about 16 × 14.5 × 14 cm in size and was diagnosed as hepatocellular carcinoma (HCC). TACE was performed twice by injecting 20 mL iodized oil with 5-fluorouracil (500 mg), epirubicin (30 mg), and hydroxycamptothecin (10 mg) through the hepatic artery, followed by injection of gelatin sponge particles. Post-TACE CT scan revealed densely deposited lipiodol inside the tumor (Fig. 1a). After that, the patient did not receive any further treatment and still suffered from anemia and fatigue for the recent years. She had no history of liver cirrhosis or chronic hepatitis virus infection, and her menses is normal without excess of menstrual blood loss. Twelve years ago, the patient was diagnosed with retroperitoneal tumor, which was about 8 × 6 × 5 cm in size and was removed completely. Pathological examination confired the diagnosis of retroperitoneal paraganglioma, with no lymph node metastasis. The patient was followed up for 5 years with no distant metastasis or anemia, then she was lost to follow-up until liver metastasis was detected.

**Fig. 2:**
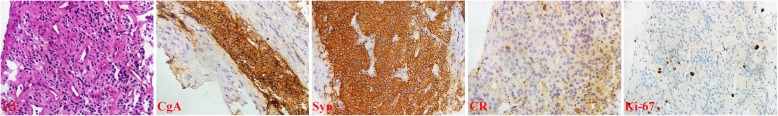
Histological and immunohistochemical examination of the liver biopsy specimens

On admission, CT scan revealed a huge solid hypodense mass in the liver (Fig. 1b), almost the same size (15 × 14 × 14 cm) as 5 years ago. The lesion showed a strong heterogeneous enhancement in the arterial phase (Fig. 1c) and was hypodense during the portal phase (Fig. 1d). Angiography demonstrated a round hypervascular mass with many feeding arteries (Fig. 1e, f). Laboratory tests revealed that hemoglobin was 74.2 g/L (normal range 110–160 g/L), hematocrit was 25.8% (normal range 33–51%), mean corpuscular volume was 77 fL (normal range 82–95 fL), mean corpuscular hemoglobin was 22.1 pg (normal range 27–31 pg), lactate dehydrogenase was 86 U/L (normal range 135-225 U/L), and superoxide dismutase was 92 U/ml (normal range 129-216 U/ml). Serum tumor marker of alpha-fetoprotein, carcinoembryonic antigen, and carbohydrate antigen 19–9 were all within normal ranges. Colonoscopy was performed and revealed normal mucosa from the rectum to the cecum. Ultrasound-guided biopsy of the liver tumor was undertaken. Histological examination of the liver biopsy specimens revealed nests of uniform neoplastic cells with vacuolated and eosinophilic cytoplasm embedded in fibrous septa with capillary vessels (Fig. 2). Immunohistochemical examination (Fig. 2) showed that the tumor was strongly stained positive for chromogranin A (CgA) and synaptophysin (Syn), but negative for calretinin (CR). The Ki-67 labeling index was <1%. The histology of the liver tumor was in accordance with the primary retroperitoneal paraganglioma, and the diagnosis of hepatic metastatic paraganglioma was confirmed. Therefore, according to the AJCC cancer staging manual (8th edition), the staging of the primary retroperitoneal paraganglioma should be T2N0M1b. Because the tumor remained stable, the patient refused to receive radiotherapy and other treatment and was followed up for another 1 years without evidence of deterioration (Additional file 1: Figure S1).

## Discussion and conclusions

Paragangliomas, also known as chemodectomas, are rare tumors that constitute less than 0.5% of all neoplasms. They arise from chemoreceptor tissue which is thought to be of mesodermal origin with mesoblastic and neural components, and most commonly locate at the bifurcation of the common carotid, the jugular foramen, aortic arch, and retroperitoneum [9]. In the third edition of the World Health Organisation (WHO) classification from 2004, paragangliomas were classified as malignant or benign on the basis of metastasis, and “malignancy” is defined as the development of metastatic lesions in non-chromaffin tissues [10]. It’s now known that all paragangliomas have some metastatic potential, and up to 35% of patients are reported to have metastases. Therefore, the qualifiers of ‘benign’ or ‘malignant’ are no longer advocated according to the latest 2017 WHO classification and have been replaced by a concept of metastatic paraganglioma [10].

In 2017, a staging system for paragangliomas was first introduced in the 8th Edition of the AJCC Cancer Staging Manual [11]. The size of the primary tumor (≥5 cm) was identified as a prognostic risk factor of developing metastases, and extra-adrenal location or invasion was also recognized as a negative prognostic factor [11–13]. Patients with these risk factors deserve lifelong follow-up. Short-term follow-up may not accurately reflect the malignant potential of paraganglioma since a mean time of 10.3 years for the appearance of metastasis has been reported after resection of primary paraganglioma [14]. Until now, there are over 120 reported cases of metastatic paraganglioma in English literature, and paragangliomas arising in sites below the diaphragm are reported to metastasize more frequently than those sited above the diaphragm [15]. The most common distant metastatic sites include lymph nodes, lung and bone. Other organs which may be involved are the thyroid, kidney and pancreas. Metastasis to liver is extremely rare.

CT and angiography complement one another in diagnosing metastastic paraganglioma. The homogenous density of the tumor and surrounding structures are due to extensive capillary beds associated with the tumor [14], and the vascular stroma of the tumors accounts for the radiologic sign of an intense flush or pooling phase seen during arteriography [15]. Since paragangliomas are characterized by extreme vascularity and intimate relationship with major blood vessels, fine-needle biopsy is usually not recommended due to the perceived high risk for bleeding. However, because of the varieties of hypervascular hepatic lesions [16], definite diagnosis still relies on histopathological examination.

Paragangliomas are usually non-functioning and present as painless masses. However, severe anemia has been noted in quite a few patients with metastatic paragangliomas [17, 18]. Some researchers regard anemia to be a marker of persistent tumor activity, since hemoglobin will rise after resection of paraganglioma and fall again with the appearance of metastases [18]. Sweet et al. described the anemia as hypochromic and microcytic with iron studies consistent with iron deficiency [19], but it’s still under controversy [20]. It’s postulated that paraganglioma may produce some substance that interferes with erythropoietin production, and depressed serum erythropoietin level has been demonstrated in some patients [17]. Though palliation of sever anemia was attained in patient by injection of nandrolone decanoate [17], the pathogenesis of paraganglioma associated anemia is still to be clarified.

Because of the slow growth rate of paraganglioma, even metastatic tumors are compatible with a prolonged survival [17, 19]. However, curative or palliative treatment should be considered to control symptoms, improve life quality, reduce complications and prolong survival. Unlike the management of HCC, which is framed within standardized protocols [21], at present there is no clinical guideline or standardised protocol for the treatment of metastatic paraganglioma due to the rarity of the disease and the lack of prospective studies [22]. Surgical resection is still effective if the metastasis can be excised totally, but it is usually not possible due to the extension of metastases. For inoperable cases with progressive or symptomatic disease, there are various palliative treatment options, such as radiotherapy, radiofrequency, or cryoablation, as well as TACE and systemic therapies including chemotherapy or molecular targeted therapies [4, 22]. Debulking or cytoreductive surgery are not recommended because the overall value of them for metastatic paraganglioma remains uncertain [22]. Recent studies demonstrate that peptide receptor radionuclide therapy (PRRT) achieves worthwhile clinical and biochemical responses for metastatic or inoperable paragangliomas, with substantial symptomatic relief and low toxicity [22], but further randomised controlled trials are required to definitively establish the role of PRRT in the treatment of these diseases.

Though paragangliomas rarely metastasize to live, physicians should be aware of this posibility. Serious evaluation of the clinical features and biological behavior as well as careful follow-up is necessary, because the number of liver metastasis reported to date is too small and long-term follow-up is still lacking. Early detection and prompt treatment of metastastic paragangliomas are crucial for controling symptoms and improving survival. Though liver metastasis failed to achieve complete response or partial response to TACE treatment, it remained stable without progression. Further studies are needed to clarify the pathogenesis of paraganglioma associated anemia and effective therapies are expected for control of metastasis.

## Additional file

Additional file 1: The CT scan of the liver tumor during last follow-up. (TIF 3038 kb)

## Data Availability

Not applicable to this article as no datasets were generated or analyzed.

## Abbreviations

CgA: chromogranin A
CR: Calretinin
Syn: Synaptophysin
TACE: Transarterial chemoembolization
