# Correction to: Hepatic metastatic paraganglioma 12 years after retroperitoneal paraganglioma resection: a case report

**DOI:** 10.1186/s12876-019-1068-z

**Published:** 2019-08-26

**Authors:** Zhao-Ru Dong, Yan-Ni Xia, Yue-Yi Zhao, Rui Wu, Kai-Xuan Liu, Kai Shi, Lun-Jie Yan, Cheng-Yu Yao, Yu-Chuan Yan, Tao Li

**Affiliations:** 1 Department of general surgery, Qilu Hospital, Shandong University, Jinan, 250012 People’s Republic of China; 2 Department of Operating Room, Qilu Hospital, Shandong University, Jinan, 250012 China; 3 Department of Clinical Laboratory, Shandong First Medical University, Jinan, 250012 China

**Correction to: BMC Gastroenterology**

**https://doi.org/10.1186/s12876-019-1061-6**

Following publication of the original article [1], the author reported that their family name was misspelled. The details are as follows:

Incorrect name in the original article:

Rui-Yi Zhao.

Correct name:

Yue-Yi Zhao.

The original article has been corrected.
