# Supplementary figures and images for: Hepatic metastatic paraganglioma 12 years after retroperitoneal paraganglioma resection: a case report
Source: BMC Gastroenterol. 2019 Aug 8;19:142. doi: 10.1186/s12876-019-1061-6 (PMC6688228; doi:10.1186/s12876-019-1061-6)

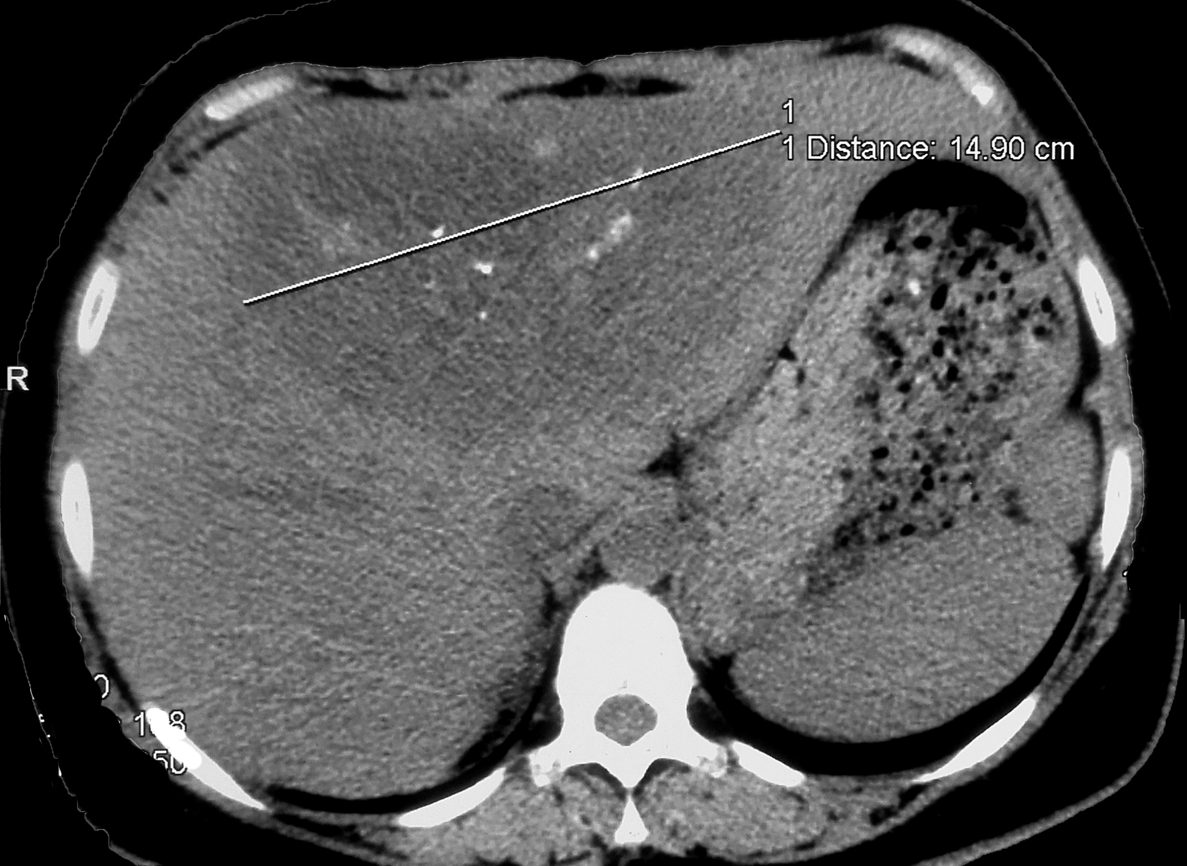

Supplement: Supplementary file 1 — The CT scan of the liver tumor during last follow-up. (TIF 3038 kb) [file 12876_2019_1061_MOESM1_ESM.tif]
